# The influence of hot isostatic pressing on precipitates, mechanical properties of Mg-12Gd-0.8Zn-0.4Zr (wt.%) alloy manufactured by sand casting

**DOI:** 10.1038/s41598-024-60309-x

**Published:** 2024-04-24

**Authors:** Dan Wang, Chao Ma, Ye Chen, Li Gu, Yangyang Yu

**Affiliations:** 1 Tianjin Renai College, Tianjin, 0086 CHN China; 2 State Key Laboratory of Engines, Tianjin University, Tianjin, 0086 CHN China

**Keywords:** Mg alloy, Hot isostatic pressing, Precipitates, Mechanical properties, Strengthening mechanism, Mechanical properties, Metals and alloys

## Abstract

Microstructures and mechanical properties of Mg-12Gd-0.8Zn-0.4Zr (GZ1208K, wt.%) alloy under different treatments (as-cast: signed as nonHIP-GZ1208K, hot isostatic pressing (HIP): signed as HIP-GZ1208K) were characterized. Based on microstructure characterization, two prismatic precipitates, β′ and β_1_ precipitates, and one basal precipitate, γ′ precipitate, formed in both of nonHIP-GZ1208K and HIP-GZ1208K alloy. According to analysis, the area number density and the size of β′ precipitate could be adjusted through HIP treatment. The area number density of β′ precipitate increased after HIP treatment when aged at 32 h, and the size of β′ precipitate refined in both of the HIP-GZ1208K alloy aged at 8 h and 32 h. Except the influence of HIP treatment on microstructures, the ultimate tensile strength (UTS) and elongation of nonHIP-GZ1208K alloy also improved after HIP treatment. The UTS of the GZ1208K alloy aged at 8 h increased from 348 MPa (nonHIP-) to 371 MPa (HIP-) and the elongation increased from 2.6% to 4.7%. The density of the nonHIP-GZ1208K alloy increased after HIP treatment, that is to say the casting defects could be eliminated and the compactness of microstructures could be increased under the high pressure of HIP treatment.

## Introduction

Since Magnesium (Mg) alloy has some outstanding properties, such as low density, high specific strength and good recyclability, it usually substitutes iron alloy or aluminium alloy in automotive and aerospace fields for light weight. Recent years, Mg-RE alloys, especially Mg-Gd-Y/Nd^1–4^ and Mg-Y-Nd^5^, obtain more attention because of their excellent room-temperature and high-temperature mechanical properties^6–10^, and these alloys have got applications as the key components in airspace fields, such as satellite support, missile case, and brake wheels of undercarriage. Nowadays, the requirements of aeronautical facilities and weapons are improving, for increasing their operational capabilities, they must have faster speed, longer range and higher accuracy, but these characteristics are limited by the mechanical properties of Mg alloys. According to the increasing standards for the aeronautical components, higher strength with good elongation of Mg alloys is necessary to meet the application requirements. Based on previous studies^11–15^, alloying is one of the most efficient methods to enhance the mechanical properties of Mg alloys and the common alloying elements are Gd and Zn element^16–18^. In Mg-12Gd-0.8Zn-0.4Zr (GZ1208K, wt.%) alloy^19^, the yield strength (YS) of sand-cast GZ1208K alloy is 270 MPa in T6 condition, which is almost the highest YS in sand-cast Mg alloys, and its elongation (EL) is 2.6%, which has more balanced properties (combined strength with elongation) compared to other high strength Mg alloys^1–5^. Therefore, sand-cast GZ1208K alloy is selected as research object in this study.

Based on the consideration of manufacturability, sand casting is usually used for moulding aerospace components, especially those ones which have complex structures and need small amount. But in fact, sand-cast alloys usually contain solidification defects (shrinkage porosity, microcrack), which are harmful to the mechanical properties of Mg alloys. Hot isostatic pressing (HIP), as one of the most effective technologies for eliminating the shrinkage porosity and microcracks in the cast alloys, has been used for improving mechanical properties in many investigations^20–27^. Ceschini^24^ reported that the ratio of total defects area to cross-section area decreases in the A356 alloy (from 3% to 0.1%) and A204 alloy (from 2% to 0.5%) after HIP process. Moreover, compared with non-HIP alloy, the defect sizes in both two HIP processed A356 and A204 alloys also decrease. Based on Xu’s report^25^, the cIosure mechanism of the shrinkage cavity is plastic deformation induced by isostatic pressure. Zheng et al.^26^ studied the effect of HIP on the closure process of casting pores in Ni-based super alloy. They found that the casting pores are healed due to the plastic flow when γ formed. In Sheng’s study^27^, compared with non-HIPed RS (rapidly solidify)-alloy, the strength of RS Ni-33Al-28Cr-5.7Mo-0.3Hf (at.%) alloy after HIP treatment increases at high temperature. Its YS value enhances from 390 to 510 MPa at 1273 K. Therefore, the HIP technology has positive influence on reducing defects and is beneficial to the mechanical properties of the cast alloy. However, very few attention has been paid to the effect of HIP on the mechanical properties of sand-cast Mg-Gd-Zn alloy.

When the solution-treated Mg-Gd-Zn alloy is aged at a lower temperature, the solubility of Gd and Zn in the Mg matrix reduces and the precipitates form in the alloys. As reported in the previous work^28^, β′ precipitates, β_1_ precipitates and γ′ precipitates can be observed in the aged GZ1208K alloy. The size, species and area number density of precipitates are influenced by many factors, such as alloying element, ageing temperature and time^1–5,11,15–17^. Different size, species and area number density of precipitates attribute to different mechanical properties of Mg alloy. Therefore, the precipitates are important for developing high-strength Mg alloys. However, the effect of HIP on the precipitates of Mg-Gd-Zn alloy has not been reported. Hence, in this paper, sand-cast GZ1208K alloy is selected as studied object, and its microstructure, precipitate behavior and strengthening mechanism in non-HIPed and HIPed GZ1208K alloy are studied. This work may have certain reference value for the application of HIP treatment on Mg-Gd-Zn alloy.

## Experimental

Mg-12Gd-0.8Zn-0.4Zr (GZ1208K, wt.%) alloy was prepared by pure Mg and Zn, Mg-87 wt.% Gd and Mg-30 wt.% Zr master alloys in an electric resistance furnace under CO_2_ and SF_6_ protect gas. The volume ratio of the protect gas of CO_2_ and SF_6_ was 99: 1. After the alloy thoroughly melted, it was poured into a sand mold which had been preheated to 200 °C for removing moisture and increasing the mobility of the melt alloy when casting, and then, the sand mold naturally cooled in air. The actual chemical composition of the ingot was Mg-11.99Gd-0.73Zn-0.35Zr (wt.%), which detected by an inductively coupled plasma atomic emission apectroscopy (ICP-AES) analyzer (Perkin-Elmer, Plasma 400).

The as-cast ingot was divided into three samples with 80 mm in length, 20 mm in width and 20 mm in height. One sample was HIP-treated at 120 MPa and 515 °C for 3 h (signed as HIP-GZ1208K), the second sample as a comparison without any treatment (nonHIP-GZ1208K), the last one was solution-treated at 515 °C for 6 h, which was used for studying the effect of pressure of HIP on the microstructure. HIPed and nonHIPed GZ1208K samples solution-treated at 530 °C for 18 h, then quenched into hot water (~ 90 °C). The solution-treated HIP-GZ1208K and nonHIP-GZ1208K alloys were signed as HIP-GZ1208K-T4 and nonHIP-GZ1208K-T4 in this paper. Ageing treatment followed by solution treatment was treated at 225 °C in an oil bath for different times. The nonHIP-GZ1208K-T4 alloy and HIP-GZ1208K-T4 alloy after ageing treatment were named as nonHIP-GZ1208K-T6 and HIP-GZ1208K-T6 in this study. Vickers hardness testing was used to measure ageing hardness, its testing load was 49 N and dwelling time was 15 s. Tensile testing was carried out on a Zwick/Roell-100 kN material test machine (Zwick USA, Kennesaw, GA) with a strain rate of 0.5 × 10^–3^ s^−1^ at room temperature. The standard size of the gauge section of the samples used for tensile test were 15.0 × 3.5 × 2.0 mm. Three tensile samples were used in each condition for data accuracy.

Microstructures were observed in optical microscope (OM, Zeiss Axio observer), scanning electron microscope (SEM, FEI Nova Nano SEM 230) equipped with Energy dispersive X-ray spectrometer (EDS) and transmission electron microscopy (TEM, FEI Tencai g2 20). OM and SEM samples prepared as follows: firstly, grinded by 360 #, 800 #, 1200 # and 3000 # sand paper in sequence; secondly, polished by diamond polishing paste; finally, the polished samples etched by picric acid. TEM samples used 360 # and 800 # sand paper for reducing its thickness to ~ 50 μm, then used ion polishing system for further reduction, finally, preserved the prepared TEM sample in vacuum vessel for electron microscopy observation. The length and the width of the β′ precipitate were the average values based on over 200 β′ precipitates. The direction of the length of the β′ precipitate was along $\left[ {01\overline{1}0} \right]_{\alpha }$ and the direction of the width of the β′ precipitate was along $\left[ {11\overline{2}0} \right]_{\alpha }$. Ipp software was used for measuring the sizes of the β′ precipitate along the directions of length and width, and then, calculated the average values of length and width through 200 β′ precipitates. The area number density of β′ precipitate was the average of five values which obtained through dividing number of precipitates into area of TEM image. VEGA 3 TESCAN was used to obtain fracture morphologies of alloys, in order to gain more details, two modes, SE and BSE mode, were selected to observe the fracture morphology.

## Results

### Microstructures of as-cast and solution-treated alloy

**Figure 1:**
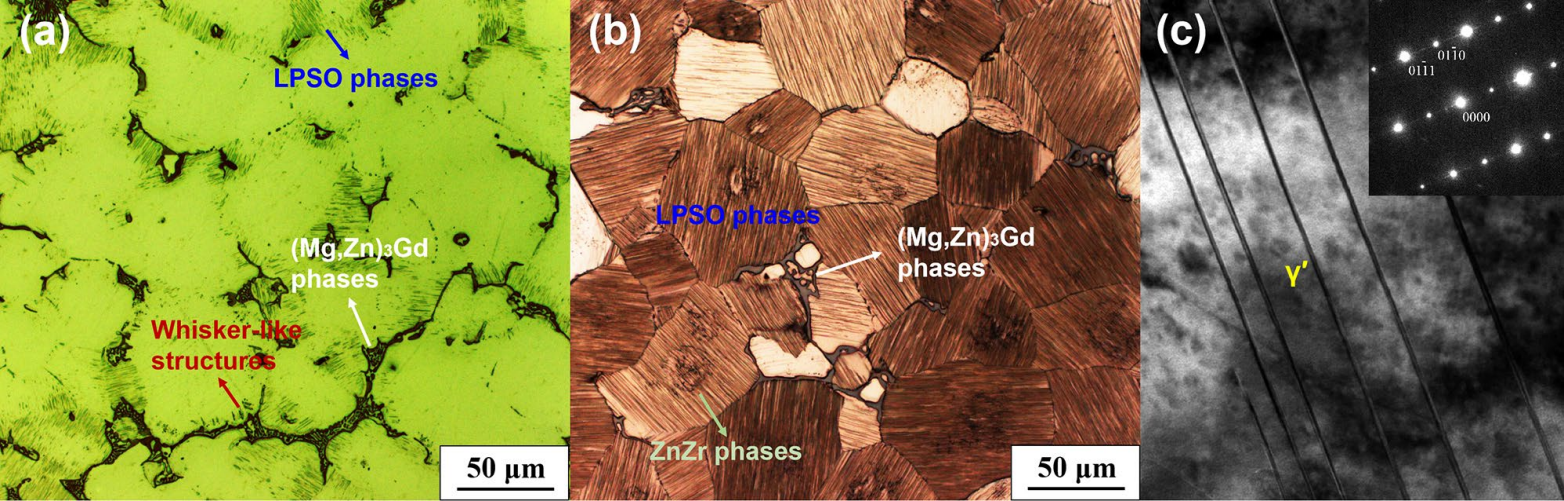
Optical images showing microstructures of alloys: (**a**) nonHIP-GZ1208K; (**b**) HIP-GZ1208K; (**c**) TEM bright field image showing LPSO phases from (**b**).

**Figure 2:**
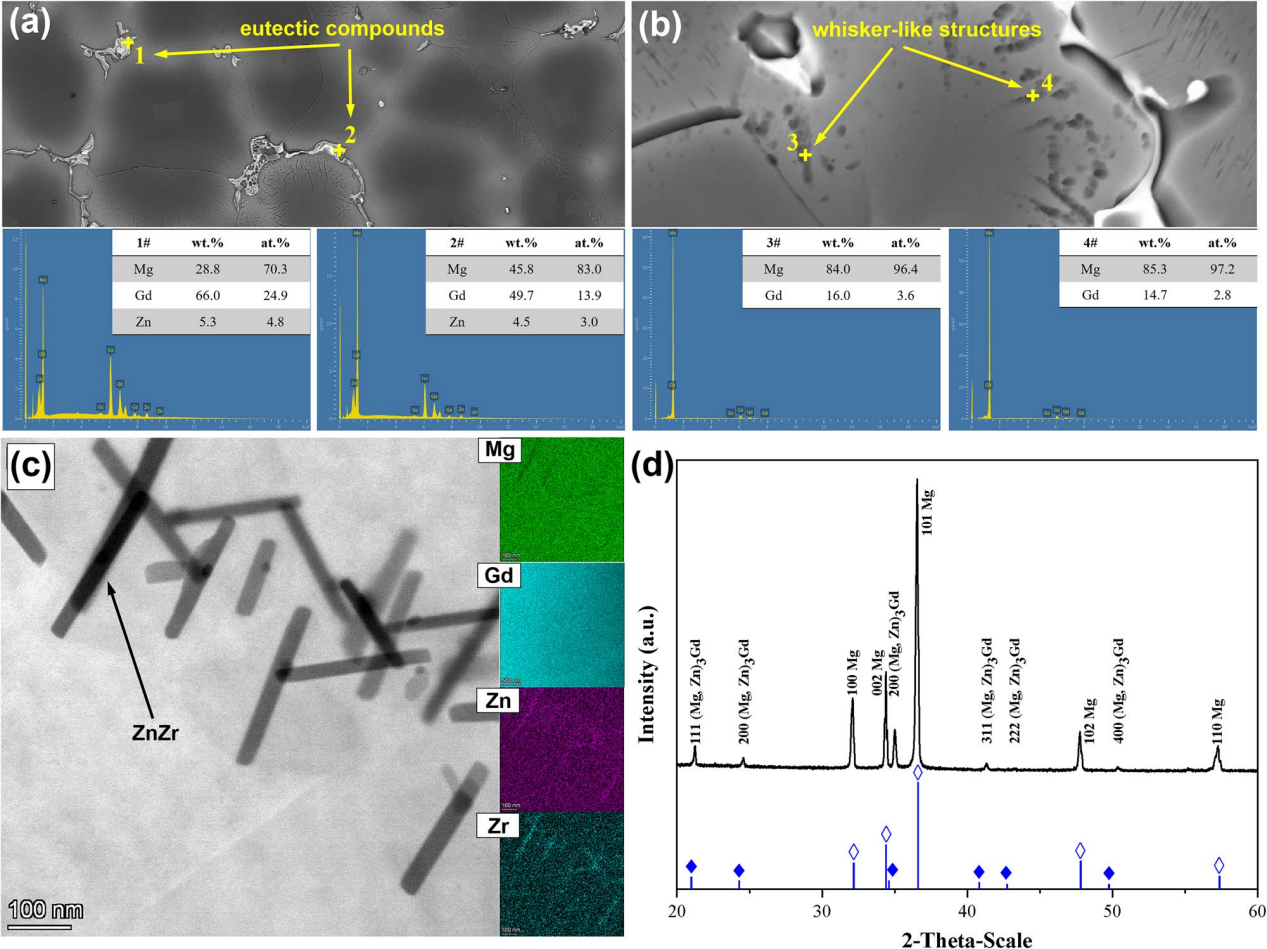
The composition of (**a**) eutectic compounds, (**b**) whisker-like structures and (**c**) ZnZr phases; (**d**) the XRD of the nonHIP-GZ1208K alloy.

Figure 1 shows microstructures of nonHIP-GZ1208K and HIP-GZ1208K alloys. NonHIP-GZ1208K alloy (Fig. 1 a) consists of α-Mg, eutectic compounds on the grain boundaries, lamellar structure and whisker-like structure around the grain boundaries. According to the EDS analysis (Fig. 2a), the eutectic compounds are composed of Mg, Gd and Zn element and the atomic ratio of Mg + Zn and Gd is about 3:1. Based on the XRD data (Fig. 2d) and the previous studies^19,28^, the eutectic compounds are identified as (Mg, Zn)_3_Gd phases. The lamellar structure is identified to be γ′ basal precipitate and this can also be observed in other Mg-Gd-Zn alloys^29–32^. The composition of whisker-like structure is Mg and Gd as showed in Fig. 2b, and Mg is the dominant element in this structure. From the XRD result, there are no peaks reflect the whisker-like structures, thus, it can be explained that the whisker-like structure is a solid solution generated by Gd segregation in the α-Mg matrix, which has been reported in our previous study^28^. In HIP-GZ1208K alloy, besides α-Mg and (Mg, Zn)_3_Gd compounds on the grain boundaries, lots of lamellar structures and black clustered particles within the grains are also observed in Fig. 1 b. The lamellar structures are γ′ basal precipitates^29–31^, and these black clustered particles with needlelike shape (Fig. 2c) are ZnZr phases based on the EDS mapping and Gao’ report^34^. According to Fig. 1 a and b, it can be seen that after HIP treatment, the volume fraction of eutectic compounds slightly decreases, from 7.8% to 7.5%, while the number density of γ′ basal precipitate rapidly increases within the grains. Figure 1 c presents the high magnification of γ′ basal precipitates in Fig. 1 a and b, the ratio of length over thickness of γ′ basal precipitates are over 500 : 1, and as reported by Zhang^33^, the γ′ basal precipitates are the structural unit of LPSO phase. The average grain sizes of nonHIPed and HIPed GZ1208K alloy are ~ 85.8 μm and ~ 81.4 μm, respectively. Grains grow slightly under HIP treatment.

**Figure 3:**
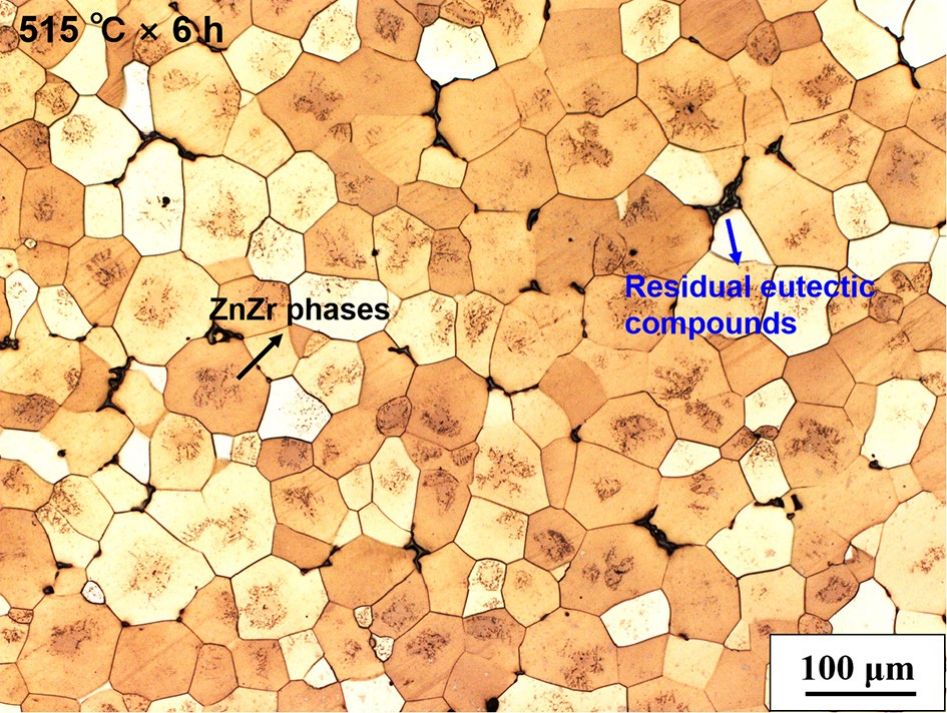
Optical image showing microstructure of solution-treated GZ1208K alloys. The solution treatment is 515 °C × 6 h.

Figure 3 shows microstructure of GZ1208K alloy solution-treated at 515 °C for 6 h. Compared with HIPed alloy (515 °C × 120 MPa × 3 h, Fig. 1b), only residual eutectic compounds and ZnZr phases are observed and no LPSO phases precipitate in the alloy even at a longer solution-treated time (515 °C × 6 h). Precipitation of LPSO phases usually depends on stacking faults^30,31^ in grains. Under high pressure, 120 MPa in HIP treatment, the stacking faults are easy to form, thus, dense LPSO phases precipitate within grains as showed in Fig. 1 b.

**Figure 4:**
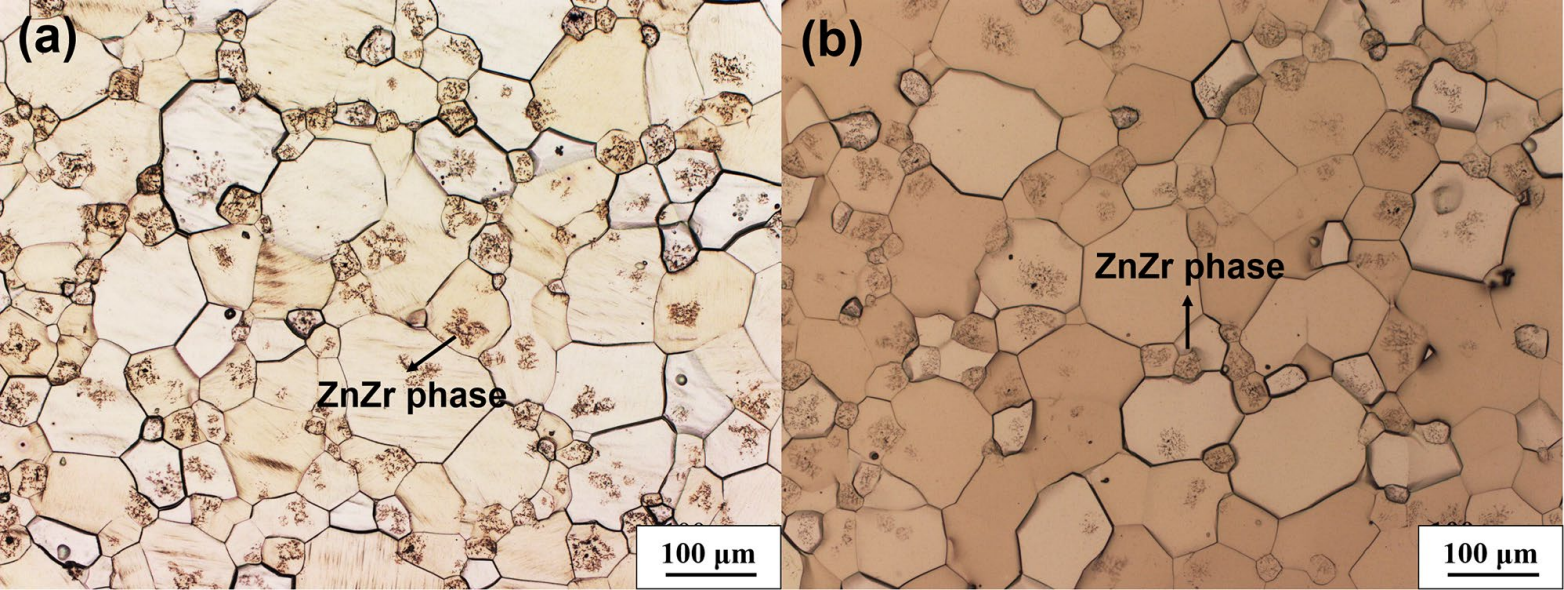
Optical images showing microstructures of (**a**) nonHIP-GZ1208K-T4 alloy and (**b**) HIP-GZ1208K-T4 alloy.

Figure 4 shows OM images of microstructures in nonHIP-GZ1208K and HIP- GZ1208K alloy after solution treatment. It can be seen that the eutectic compounds almost dissolve into α-Mg in the nonHIP-GZ1208K-T4 alloy, and meanwhile, ZnZr phases precipitate within grains. In the HIP-GZ1208K-T4 alloy, both of the eutectic compounds and LPSO phases dissolve into the matrix, while more ZnZr phases form within grains. The grain sizes of nonHIP-GZ1208K-T4 and HIP-GZ1208K-T4 alloy are ~ 89.5 μm and ~ 91.8 μm, respectively. Both of them slightly grow up compared with nonHIPed and HIPed GZ1208K alloy.

### Age hardening response

**Figure 5:**
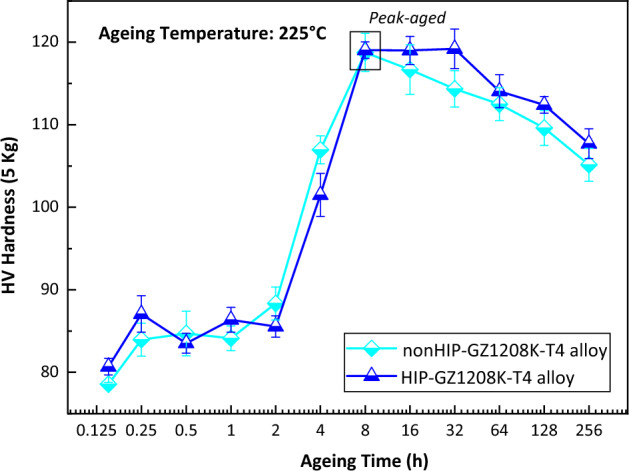
Ageing curves of nonHIP-GZ1208K-T4 and HIP-GZ1208K-T4 alloys after aged at 225 °C.

Figure 5 shows age hardening curves of nonHIP-GZ1208K-T4 and HIP-GZ1208K-T4 alloy after aged at 225 °C. It is obvious that ageing hardness of HIP-GZ1208K-T6 alloy is similar with nonHIP-GZ1208K-T6 alloy at underaged stage, while at overaged stage, the ageing hardness of HIP-GZ1208K-T6 alloy is slightly higher than nonHIP-GZ1208K-T6 alloy. The peak hardness of the two alloys are 119 HV when aged for 8 h, and this hardness value maintains constant until aged for 32 h in the HIP-GZ1208K-T6 alloy, while in the nonHIP-GZ1208K-T6 alloy, its hardness directly descents after peak-aged for 8 h. The age hardening ability (△HV, HV_peak hardness_—HV_solution-treated hardness_) of nonHIP-GZ1208K-T6 and HIP-GZ1208K-T6 alloy are 39.78 HV and 38.15 HV, respectively, the difference between them is small. Therefore, HIP treatment has little influence on age hardening ability and time to peak hardness.

### Mechanical property

**Figure 6:**
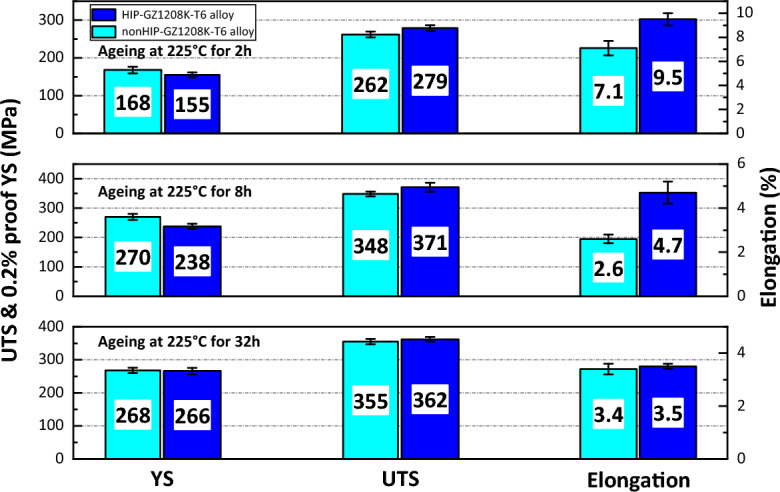
Mechanical properties of nonHIP-GZ1208K-T6 and HIP-GZ1208K-T6 alloy at different ageing times at 225 °C.

Figure 6 shows mechanical properties of nonHIP-GZ1208K-T6 and HIP-GZ1208K-T6 alloy aged for different conditions: aged at 225 °C for 2 h, 8 h & 32 h. As aged for 2 h, the yield strength (YS), ultimate tensile strength (UTS) and elongation of HIP-GZ1208K-T6 alloy are 155 MPa, 279 MPa and 9.5%, respectively. Compared with the nonHIP-GZ1208K-T6 alloy, YS reduces—13 MPa, while UTS and elongation enhance + 17 MPa and + 2.4%, respectively. When aged for 8 h, the HIP-GZ1208K-T6 alloy still has lower YS value and higher UTS and elongation value compared with nonHIP-GZ1208K-T6 alloy aged for 8 h. The reduction of YS is—32 MPa, and the enhancement of UTS and elongation are + 23 MPa and + 2.1%, respectively. Continuing aged for 32 h, the HIP-GZ1208K-T6 and nonHIP-GZ1208K-T6 alloy show comparable YS, UTS and elongation value, the differences between the two alloys of YS, UTS and elongation are reduced. Further analysis discovers that the elongations of the HIP-GZ1208K-T6 alloy aged for different times are higher than the nonHIP-GZ1208K-T6 alloy. The higher elongations of HIP-GZ1208K-T6 alloy aged for different times illustrate that the micro-defects may be reduced and the compactness of microstructures may be enhanced by HIP treatment.

Through measurement, the density of HIP-GZ1208K alloy (1.949 g/cm^3^) is higher than that of nonHIP-GZ1208K alloy (1.942 g/cm^3^), it increases after HIP treatment. This result indicates that the HIP treatment can increase microstructure compactness and eliminate some casting defects. Casting defects as crack origins usually generate cracks and these cracks will propagate when alloys under stress. Thus, the elimination of casting defects is beneficial to enhance strength and elongation of alloys. From Fig. 6, it can be seen that the UTS and elongation of HIP-GZ1208K-T6 alloy with different ageing times are higher than nonHIP-GZ1208K-T6 alloy, and this can owe to HIP treatment.

### Precipitates

**Figure 7:**
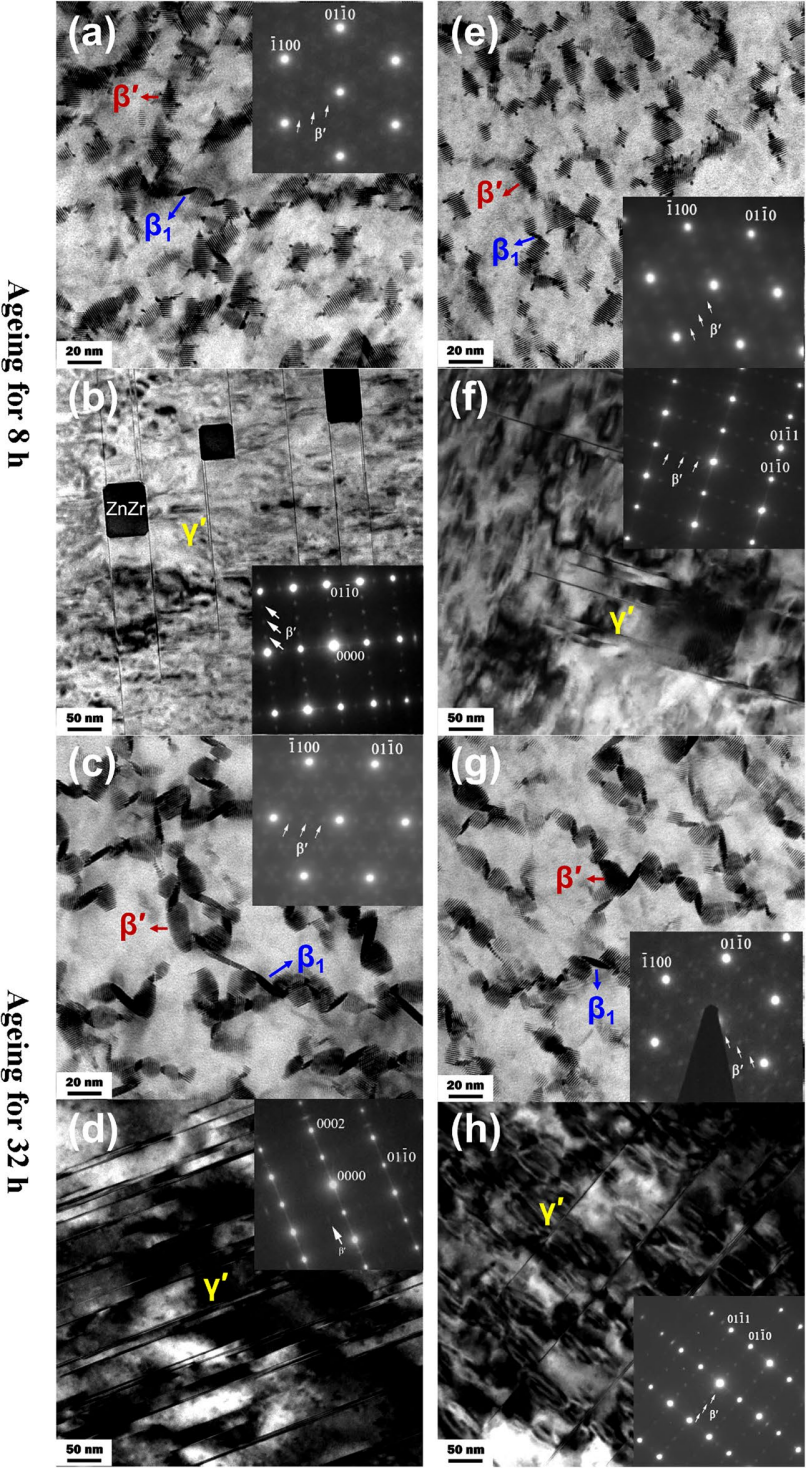
TEM BF images and corresponding SAED patterns of (**a**, **b**, **c** and **d**) nonHIP-GZ1208K-T6 alloy and (e, f, g and h) HIP-GZ1208K-T6 alloy. The ageing times in (a, b, e and f) were 8 h at 225 °C, while the ageing times in (c, **d**, **g** and **h**) were 32 h at 225 °C. The beams were parallel to [0001]_α_ direction in (a, e, c and g) and were parallel to^11–20^_α_ direction in (b, f, d and h).

Figure 7 shows TEM bright field images and corresponding SAED patterns of nonHIP-GZ1208K-T6 and HIP-GZ1208K-T6 alloy at ageing time of 8 h and 32 h. In Fig. 7 a and Fig. 7 e, two kinds of prismatic precipitates with oval and parallelogram shape form in both nonHIP-GZ1208K-T6 and HIP-GZ1208K-T6 alloy at ageing time of 8 h. From SAED patterns in Fig. 7 a and Fig. 7 e, the diffraction points at 1/4 $\left\{ {01\overline{1}0} \right\}_{\alpha }$, 1/2 $\left\{ {01\overline{1}0} \right\}_{\alpha }$ and 3/4 $\left\{ {01\overline{1}0} \right\}_{\alpha }$ demonstrate that the prismatic precipitates with oval shape are β′ precipitates^19,28,32^. Another prismatic precipitates with parallelogram shape are β_1_ precipitates which have been detailed investigated by Nie^35^. Besides prismatic precipitates, γ′ basal precipitates are also observed in both of alloys aged for 8 h (shown in Fig. 7b,f). When aged for 32 h, β′ and β_1_ prismatic precipitates grow and coarsen compared with alloys aged for 8 h and the density of γ′ basal precipitates increases.

Table 1 summarizes the length (L) and width (W) of β′ and β_1_ precipitate in nonHIP-GZ1208K-T6 and HIP-GZ1208K-T6 alloy at ageing time of 8 h and 32 h. The sizes of β′ and β_1_ precipitate in HIP-GZ1208K-T6 alloy are more fine than those in nonHIP-GZ1208K-T6 alloy. Table 2 lists the area number densities of β′ precipitate in nonHIP-GZ1208K-T6 and HIP-GZ1208K-T6 alloys. When aged for 8 h, compared with nonHIP-GZ1208K-T6 alloy, the HIP-GZ1208K-T6 alloy has a lower area number density of β′ precipitate (- 0.15 × 10^15^ m^-2^) and a higher area number density of β_1_ precipitate (+ 0.10 × 10^15^ m^-2^). After aged for 32 h, inversely, compared with nonHIP-GZ1208K-T6 alloy, the HIP-GZ1208K-T6 alloy has a higher area number density of β′ precipitate (+ 0.36 × 10^15^ m^-2^) and a lower area number density of β_1_ precipitate (- 0.11 × 10^15^ m^-2^). In the nonHIP-GZ1208K-T6 and HIP-GZ1208K-T6 alloy, compared 32 h with 8 h, the area number densities of β′ precipitate decrease—0.81 × 10^15^ m^-2^ and—0.30 × 10^15^ m^-2^, respectively, while the area number densities of β_1_ precipitate increase + 2.59 × 10^15^ m^-2^ and + 2.34 × 10^15^ m^-2^, respectively.

**Table 1:** The length (L) and width (W) of β′ and β_1_ precipitates in nonHIP-GZ1208K-T6 alloy and HIP-GZ1208K-T6 alloy at different ageing times.

			nonHIP-GZ1208K-T6	HIP-GZ1208K-T6
β′	8 h	Length (nm)	14.04	11.89
		Width (nm)	8.83	7.65
	32 h	Length (nm)	15.37	11.76
		Width (nm)	10.11	9.13
β_1_	8 h	Length (nm)	11.27	10.11
		Width (nm)	5.34	4.61
	32 h	Length (nm)	15.86	15.07
		Width (nm)	5.77	5.28

L stood for the length along $\left[ {01\overline{1}0} \right]_{\alpha }$; W stood for the length along $\left[ {11\overline{2}0} \right]_{\alpha }$.

**Table 2:** The area number density of β′ and β_1_ precipitate in nonHIP-GZ1208K-T6 alloy and HIP-GZ1208K-T6 alloy at different ageing times.

A (× 10^15^ m^−2^)		nonHIP-GZ1208K-T6	HIP-GZ1208K-T6	Δ_HIP-nonHIP_
8 h	β′	1.65	1.50	− 0.15
	β_1_	0.64	0.74	+ 0.10
32 h	β′	0.84	1.20	+ 0.36
	β_1_	3.19	3.08	− 0.11
Δ_32h-8 h_	β′	− 0.81	− 0.30	Δ (× 10^15^ m^−2^)
	β_1_	+ 2.59	+ 2.34	

*A represents the area number density.

*Δ_HIP-nonHIP_ = A_HIP_—A_nonHIP_; Δ_32h-8 h_ = A_32h_—A_8h_.

Based on analysis above, compared with nonHIP-GZ1208K-T6 alloy, the area number density of β′ precipitate in HIP-GZ1208K-T6 alloy at ageing time of 8 h decreases by—9.1% ($grwoth\,rate\,of\,area\,number\,density = \frac{{\Delta_{HIP - nonHIP} }}{{A_{nonHIP} }}$) and the area number density of β_1_ precipitate increases by + 16.6%, as showed in Table 3, however, when at ageing time of 32 h, the area number density of β′ precipitate in HIP-GZ1208K-T6 alloy increases by + 42.8% and the area number density of β_1_ precipitate decreases by—3.4%. From Fig. 6 and Table 3, compared the nonHIP-GZ1208K-T6 alloy with the HIP-GZ1208K-T6 alloy at ageing time of 8 h, the value of ΔYS_HIP-nonHIP_ is—32 MPa with—9.1% decrease of β′ precipitate and + 16.6% increase of β_1_ precipitate. When at ageing time of 32 h, the value of ΔYS_HIP-nonHIP_ is—2 MPa with + 42.8% increase of β′ precipitate and—3.4% decrease of β_1_ precipitate in the HIP-GZ1208K-T6 alloy. According to previous studies ^28,36^, γ′ precipitates have little contribution to strength. In this study, the number density of γ′ precipitates is very small, thus their contributions to strength can be neglected. It is therefore, the improvement of ΔYS_HIP-nonHIP_ value (from—32 MPa to—2 MPa) is mainly due to the increment of growth rate of the area number density of β′ precipitate (from—9.1% to + 42.8%). Besides the area number density of β′ precipitate, the size of β′ precipitate also influences the YS value. The YS value is inversely proportional to the size of precipitates. Finer precipitates contribute to higher YS value. From Table 1, it can be seen that the size of β′ precipitate in the HIP-GZ1208K-T6 alloy is smaller than the size of β′ precipitate in the nonHIP-GZ1208K-T6 alloy. Thus, compared with nonHIP-GZ1208K-T6 alloy, small size of β′ precipitate in the HIP-GZ1208K-T6 alloy also causes the increase of ΔYS_HIP-nonHIP_ value. Hence, β′ precipitates have important effect on yield strength, and the HIP treatment as one of the common treatments can adjust the area number density and size of β′ precipitate which are important factor for improving strength.

**Table 3:** The growth rates of the area number densities of β′ and β_1_ precipitate and the values of ΔYS_HIP-nonHIP_ at different ageing times of the nonHIP-GZ1208K-T6 alloy and HIP-GZ1208K-T6 alloy.

		Growth rate of area number density	ΔYS_HIP-nonHIP_
8 h	β′	− 9.1%	− 32 MPa
	β_1_	+ 16.6%	
32 h	β′	+ 42.8%	− 2 MPa
	β_1_	− 3.4%	

*Growth rate of area number density = Δ_HIP-nonHIP_/A_HIP_.

*ΔYS_HIP-nonHIP_ = YS_HIP_—YS_nonHIP_.

### Fracture surface

**Figure 8:**
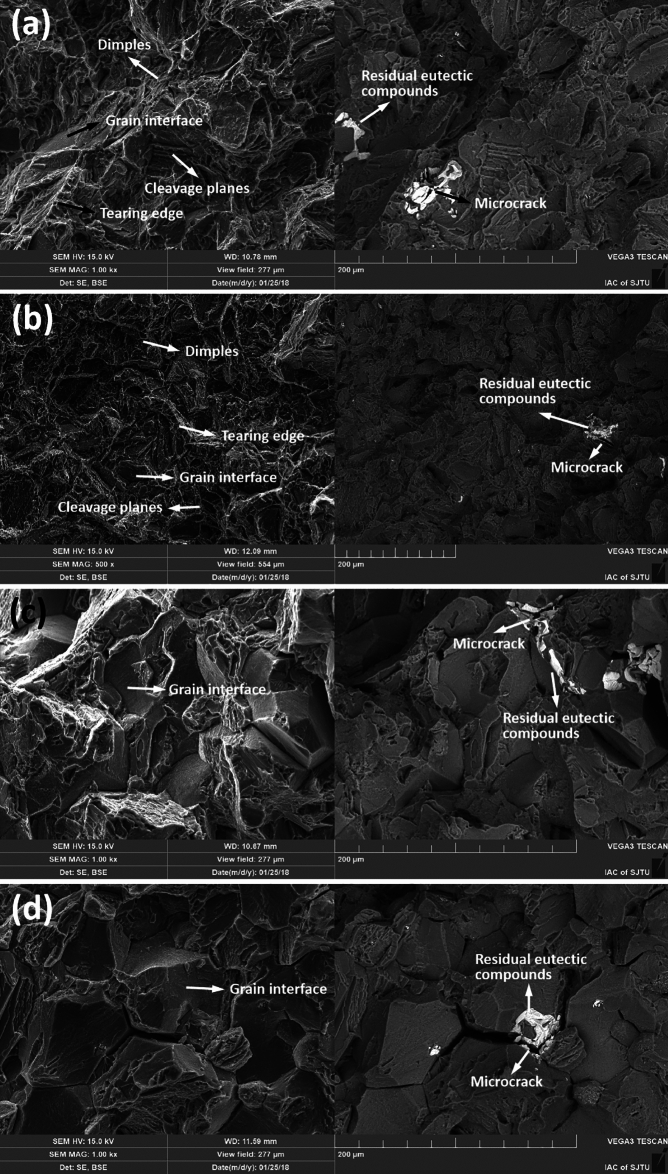
SEM images with SE (left column) and BSE (right column) mode of fracture surface of (**a**) nonHIP-GZ1208K-T6 alloy and (**b**) HIP-GZ1208K-T6 alloy at ageing time of 2 h; (**c**) nonHIP-GZ1208K-T6 alloy and (**d**) HIP-GZ1208K-T6 alloy at ageing time of 32 h.

Figure 8 shows fracture morphologies of nonHIP-GZ1208K-T6 and HIP-GZ1208K-T6 alloy at ageing time of 2 h and 32 h. From Fig. 8 a-b, lots of cleavage planes and dimples are observed in both of nonHIP-GZ1208K-T6 and HIP-GZ1208K-T6 alloy at ageing time of 2 h. The formation of cleavage plane is due to the propagation of microcracks along specific crystal planes of grains in alloy, and then these crystal planes generate separation under shear stress and leave on the fracture. Dimples are formed due to nucleation and growth of micropores, and when the micropores assemble together, alloy begins to fracture and dimples are left on the fracture surface. In Fig. 8 c-d, it can be seen that many grain interfaces appear in both of nonHIP-GZ1208K-T6 and HIP-GZ1208K-T6 alloy at ageing time of 32 h, this is due to the microcracks propagate along grain boundaries, and lead to breakage of the two alloys along grain boundaries.

## Disscusion

### Influence of HIP treatment on the β′ precipitate

According to analysis in "precipitates", when at ageing time of 8 h, the area number density of β′ precipitate in HIP-GZ1208K alloy (1.50 × 10^15^ m^-2^) is lower than the nonHIP-GZ1208K alloy (1.65 × 10^15^ m^-2^), however, when at ageing time of 32 h, the area number density of β′ precipitate in HIP-GZ1208K (0.74 × 10^15^ m^-2^) alloy is higher than the nonHIP-GZ1208K alloy (0.64 × 10^15^ m^-2^), as showed in Table 2.

As Zheng’ report ^26^, the elimination of casting defects, such as microcracks and micropores, during HIP treatment, are based on plastic flow of the materials around casting defects. The mechanism of plastic flow of the materials under high temperature and high pressure are the slippage of dislocations, then, the dislocations are expended in order to close microcracks and micropores, and the density of dislocation decreases in alloy. The temperature and pressure of HIP treatment in the study are 515 °C and 120 MPa, respectively. Dislocations in this conditions are easy to slip and the density of dislocation in the HIP-GZ1208K alloy decreases for eliminating the casting defects. According to Heng’ report ^38^, in the Mg12.6Gd1.3Y0.9Zn0.5Mn (wt.%) alloy, the existence of dislocations can promote the formation of β′ precipitate, and then the number density of β′ precipitate increases with the dislocation density. This may be due to that Gd is the important element in the β′ precipitate ^35,36^, the formation of β′ precipitates need dislocations act as channels to transport Gd elements to form short-rang ordered solute clusters which are the pre-precipitation phases of nuclei of β′ precipitates. Based on the analysis above, HIP-GZ1208K alloy has a lower dislocation density, then, the nucleation rate of β′ precipitate decreases, thus, when aged for 8 h, the area number density of β′ precipitate in the HIP-GZ1208K alloy is lower than the area number density of β′ precipitate in the nonHIP-GZ1208K alloy. Due to the lower nucleation rate of β′ precipitate in the HIP-GZ1208K alloy, the time of reaching the maximum of the area number density of β′ precipitate and the time of phase transition from β′ precipitate to β_1_ precipitate are delayed, thus, the HIP-GZ1208K alloy has a higher area number density of β′ precipitate when extending the ageing time to 32 h.

From Table 1, it can be seen that the size of β′ precipitate in the HIP-GZ1208K alloy is smaller than the size of β′ precipitate in the nonHIP-GZ1208K alloy. The determinants of the β′ precipitate size are the critical nucleation radius (γ_c_) of the β′ precipitate and growth rate of β′ precipitate. Based on the equation of the critical nucleation radius^39^, the γ_c_ is associated with interfacial energy (σ), molar volume (V) and chemical free energy of phase transition (ΔG). Due to the same component and structure of β′ precipitate in the nonHIP- and HIP- GZ1208K alloy, the σ between α-Mg and β′ precipitate and the ΔG of β′ precipitate in the two alloys are the same. The Gd concentration of β′ precipitates in the two alloys have the same value, thus, the V value of β′ precipitates in the two alloys do not change. Based on the analysis above, it can be concluded that the γ_c_ value in the nonHIP-GZ1208K alloy is the same with the γ_c_ value in the HIP-GZ1208K alloy. Therefore, the growth rate of β′ precipitate has become the main factor that determines the size of β′ precipitate. The growth rate of β′ precipitate is associated with the diffusion rate of Gd, and the diffusion rate of Gd is related with the density of dislocations, which can provide transmission channels for accelerating the Gd diffuse. Since the HIP-GZ1208K alloy has lower density of dislocation, the growth rate of β′ precipitates are limited by the reduction of dislocation density, thus, the sizes of β′ precipitates in the HIP-GZ1208K are small.

### The fracture mechanism in the nonHIP- and HIP- GZ1208K alloy

**Figure 9:**
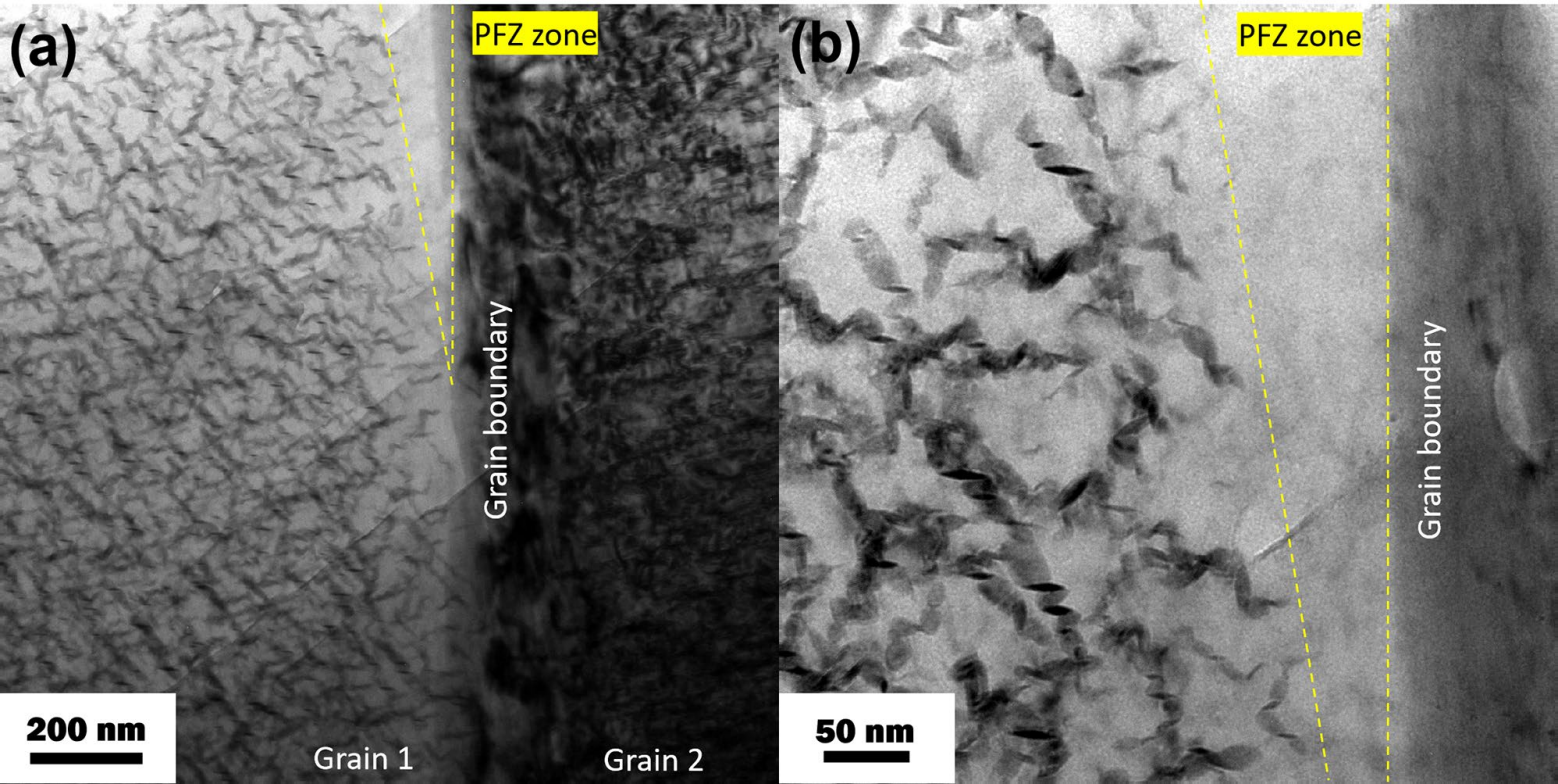
(**a**) TEM image of microstructure of grain boundary in HIP-GZ1208K-T6 alloy at ageing time of 32 h. (**b**) TEM image of (**a**) at high magnification.

According to Fig. 8, the fracture mechanism of nonHIP-GZ1208K-T6 and HIP-GZ1208K-T6 alloys are different with the ageing time. The fracture mechanism of the two alloys at ageing time of 2 h are the combination of cleavage fracture and dimple fracture, while at ageing time of 32 h, cleavage planes and dimples are seldomly found in the fracture surface, and fracture morphology is dominated by grain interfaces, thus, the fracture mechanism of the two alloys aged for 32 h are intergranular fracture. There are two possible reasons for the different fracture mechanisms. One is that the sizes of β′ precipitate in the two alloys at ageing time of 32 h are larger than that in the two alloys at ageing time of 2 h, and the area number densities of β′ precipitate in the two alloys at ageing time of 32 h are higher than the two alloys at ageing time of 2 h^19^. β′ precipitates are the main barriers in the grains of nonHIP-GZ1208K-T6 and HIP-GZ1208K-T6 alloy. Their hindering effects are associated with the size and area number density of β′ precipitate, larger size and higher area number density of β′ precipitate contribute to higher hindering effect. Thus, the grains, which possess larger size and higher area number density of β′ precipitates in the 32 h aged two alloys, have higher resistance capacity of microcrack propagation, and when the microcracks try to propagate through the grains along some special crystal planes or micropores, they are prevented in the grains, then, cleavage fracture and dimple fracture seldomly happen in the two alloys at ageing time of 32 h. The other is there have precipitation free zones (PFZs) in the grain boundaries and no β′ phases precipitate in the PFZs, as showed in Fig. 9. These PFZs are also observed by Zhang in Mg-Gd alloy ^37^. Due to lack of strengthening effect of β′ precipitates, the grain boundaries in the PFZs are easy to glide during tensile testing, and then the alloy prefers to fracture along grain boundaries and occurs intergranular fracture. In the two alloys at ageing time of 32 h, because of its higher resistance capacity of microcrack propagation in the grains, the PFZs become the weakest points and the microcracks can propagate along grain boundaries during tensile test. Therefore, lots of grain interfaces can be seen in the fracture morphologies of the two alloys at ageing time of 32 h (Fig. 8c and d). Compared SE mode (left column) with BSE mode (right column), several microcracks can be found in residual eutectic phases, which as brittle phases easily produce microcracks in nonHIP-GZ1208K-T6 and HIP-GZ1208K-T6 alloy. Therefore, the existence of residual eutectic phases promote fracture in two alloys at ageing time of 2 h and 32 h.

**Figure 10:**
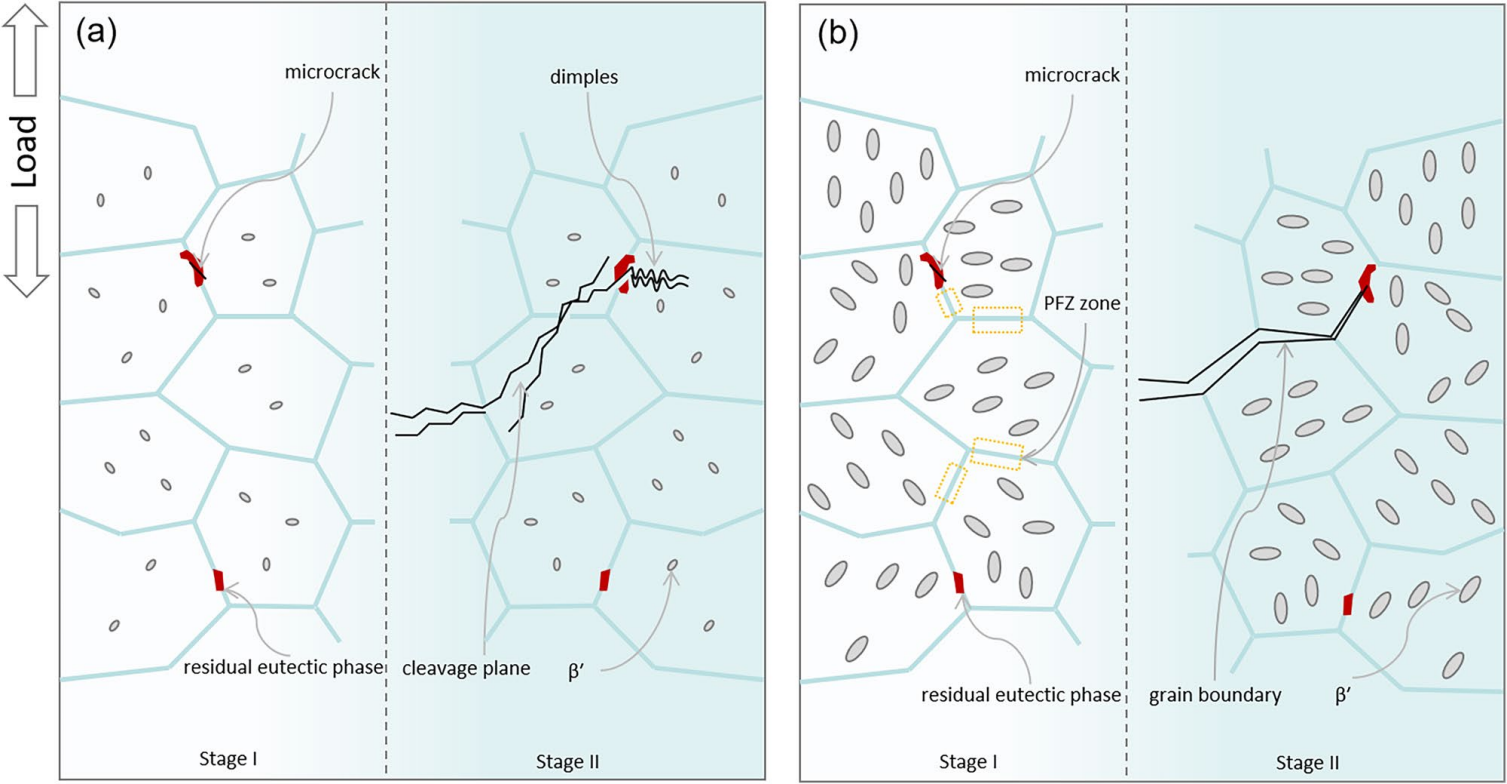
The schedule of microcrack propagation process in nonHIP-GZ1208K-T6/HIP-GZ1208K-T6 alloy at ageing time of 2 h (**a**) and 32 h (**b**). Stage I and stage II stand for the initial and late stage of the microcrack propagation, respectively.

Figure 10 shows the schedule of microcrack propagation process of nonHIP-GZ1208K-T6/HIP-GZ1208K-T6 alloy at ageing time of 2 h and 32 h. Weak points, such as residual eutectic phases and PFZs, are usually the places that microcracks prefer to generate in. At ageing time of 2 h, the microcracks may produce in the residual eutectic phases under stress, then, they propagate along specific crystalline planes within grains and leave transgranular cleavage planes in the fracture. At ageing time of 32 h, due to the microcracks have been highly hindered by the β′ precipitates which have large size and high area number density compared with the two alloys aged at 2 h, they could only propagate along grain boundaries through PFZs, and then leave grain interfaces in the fracture. Therefore, the fracture mechanism in the two alloys aged for 2 h is the mixed mode of cleavage fracture and dimple fracture and the fracture mechanism in the two alloys aged for 32 h is intergranular fracture.

## Conclusions

To analyze the effect of hot isostatic pressing (HIP) on Mg-12Gd-0.8Zn-0.4Zr (GZ1208K, wt.%) alloy, the microstructures, mechanical properties and fracture morphologies of nonHIP-GZ1208K and HIP-GZ1208K alloy are studied. The main findings are summarized as follows.

1. HIP treatment can promote the formation of γ′ basal precipitates. Compared with nonHIP-GZ1208K alloy, dense γ′ basal precipitates form within grains of HIP-GZ1208K alloy.
2. HIP treatment can reduce casting defects and increase compactness of cast GZ1208K alloy, and then the ultimate tensile strength and elongation of nonHIP-GZ1208K-T6 alloy have been enhanced after HIP treatment.
3. HIP treatment can adjust the area number density and the size of β′ precipitate which usually acts as strengthening phase in the Mg-Gd-Zn alloy and has important effect on strength.
4. The fracture mechanism is associated with the size and the area number density of β′ precipitate. Alloy with smaller size and lower area number density of β′ precipitate tends to has fracture mechanism of the combination of cleavage fracture and dimple fracture, and alloy with larger size and higher area number density of β′ precipitate tends to has fracture mechanism of intergranular fracture.

## Data Availability

The datasets generated and/or analysed during the current study are not publicly available due to technical or time limitations but are available from the corresponding author on reasonable request.
